# Specific Phenylpropanoid Oligomerization in a Neutral Environment by the Recombinant Alkaline Laccase from *Paramyrothecium roridum* VKM F-3565

**DOI:** 10.3390/biom15101437

**Published:** 2025-10-11

**Authors:** Zhanna V. Renfeld, Alexey M. Chernykh, Sofia Yu. Gorina, Boris P. Baskunov, Olga V. Moiseeva, Natalia V. Trachtmann, Shamil Z. Validov, Marina P. Kolomytseva

**Affiliations:** 1 G.K. Skryabin Institute of Biochemistry and Physiology of Microorganisms, Pushchino Center for Biological Research of the Russian Academy of Sciences, Prosp. Nauki 5, Pushchino 142290, Moscow, Russia; 2 Federal Research Center, Kazan Scientific Center of Russian Academy of Science, ul. Lobachevskogo, 2/31, Kazan 420111, Tatarstan Republic, Russiash.validov@knc.ru (S.Z.V.)

**Keywords:** alkaline recombinant laccase, *Paramyrothecium roridum*, phenylpropanoid, oligomerization, lignin depolymerization, alkaline environment

## Abstract

Fungal laccases oxidize a wide range of substrates with a diverse spectrum of subsequent non-specific free radical reactions, leading to the production of unwanted byproducts. This work describes a unique recombinant alkaliphilic laccase from *Paramyrothecium roridum* VKM F-3565 capable of performing specific oligomerization of phenylpropanoids (precursors of natural lignin and lignans) in a neutral environment, thus preventing the reverse reaction of depolymerization which occurs in an acidic environment. The recombinant alkaliphilic laccase from *P. roridum* VKM F-3565 with a specific enzyme activity of about 154.0 U/mg (in the reaction with 1 mM ABTS) was obtained using a *Komagataella phaffii* transformant with a yield of 20 ± 1.5 mg/L. The recombinant laccase had an increased degree of N-glycosylation (MW = 97 kDa), higher pH optimum in reaction with phenylpropanoids and a decreased temperature optimum, compared to the wild-type laccase. The enzyme exhibited great resistance to surfactants and the EDTA in the neutral conditions rather than the acidic ones, whereas its tolerance to mono- and divalent-metal ions was high at acidic conditions. This work demonstrates the important role of N-glycosylation of the alkaliphilic laccase of *P. roridum* VKM F-3565 in its functional activity. The presence of pH-dependent reactions makes the studied laccase attractive for the phenylpropanoid oligomerization with the production of novel oligomeric phenylpropanoid derivatives for industrial and pharmacological purposes.

## 1. Introduction

Fungal laccases have significant advantages over bacterial laccases and laccase-like enzymes due to their higher redox potential and, consequently, broad substrate specificity without mediator application [1,2]. The emergence of new technologies, carried out in neutral-alkaline environments (cellular platforms for the biosynthesis of pharmacologically and industrially valuable compounds [3,4,5,6], biosensors and biofuel cells for implantable devices [7], 3D printed biocatalytic device [8], and synthesis of C-N heteropolymer dyes [9]), promotes the search and study of biotechnologically valuable atypical fungal laccases active in such environments. However, the most typical fungal laccases are functionally active under acidic environment, what significantly complicates their usage in technological processes operating in neutral alkaline medium [1,10,11,12,13].

Currently, only a few fungi have been found to have laccases that are most active in a neutral-alkaline environment (Table 1).

There are reports about 18 alkaliphilic laccases from basidiomycetes, for ten of which the nucleotide sequences of the corresponding genes are known: *Coprinopsis cinerea* (*Coprinus cinereus*) [14,15,16,17,18,19], *Rhizoctonia praticola* [21], *Rhizoctonia solani* [20], *Pleurotus eryngii* [22], *Pleurotus sajor-caju* [23], *Pycnoporus cinnabarinus* and basidiomycete PM1 [24], *Schizophyllum commune* [25], and *Trametes versicolor* [26,27,30].

Alkaliphilic laccases were also found in ascomycetes (Table 1): *Acremonium murorum* [28], *Chrysocorona lucknowensis* [29], *Cochliobolus heterostrophus* and *Fusarium verticillioides* [30], *Melanocarpus albomyces* [32,33,34,35,36,37], *Moniliophthora perniciosa* [38], *Myceliophthora thermophile* (*Thermothelomyces thermophiles*) [39], and *Sordaria macrospora* [43]. Among the ascomycetes of the *Myrothecium* genus, only two laccases are very active in a neutral alkaline medium: laccases of *M. verrucaria* 24G [40] and *Paramyrothecium* sp. strain MM13-F2103 [42].

Recently, a great interest has been shown in the biosynthesis of oligomeric derivatives of phenylpropanoids, especially monolignols, which in turn are natural precursors of plant polymeric lignins and lignans [5,44,45]. Oligomeric derivatives of phenylpropanoids (lignans, stilbenes, flavonoids, etc.) are pharmacologically and nutrient valuable compounds with a wide range of effects on living organisms, such as cytostatic, carcinolytic, antibacterial, fungicidal, antiparasitic, antioxidant, stimulating, and adaptogenic [3,4,5,45,46]. It is technologically convenient to carry out the biosynthesis of industrially valuable compounds in cellular platforms, where the reaction occurs in a cytosol (pH 7.0–7.5) [47,48]; however, this requires enzymes that are the most active in a neutral environment.

There is a lack of knowledge in the literature on recombinant alkaliphilic fungal laccases (Table 1), concerning their activity toward phenylpropanoids, specifically their polymerization capability.

Recently, we demonstrated the production of oligomeric derivatives of phenylpropanoids using novel fungal alkaline laccases under neutral conditions [11,31,41,49]. We isolated and characterized an alkaliphilic laccase from the culture liquid of the fungus *Paramyrothecium* (*Myrothecium*) *roridum* VKM F-3565. This enzyme oxidized phenylpropanoids in the absence of mediators in a neutral alkaline medium to form trimeric derivatives, potential analogues of biologically active lignans [41]. The nucleotide sequence of a gene presumably encoding the investigated laccase was also determined. A mechanism for the high functional activity of the alkaliphilic fungal laccases in a proton-deficient environment was proposed [41].

This study focuses on the development of a heterologous expression system for the unique recombinant alkaliphilic laccase from *P. roridum* VKM F-3565. The resulting highly glycosylated laccase was thoroughly characterized, and its application and difference from the wild-type enzyme were demonstrated. The results show the potential of this laccase for developing biotechnologies for the selective production of novel oligomeric compounds that are challenging to synthesize chemically.

## 2. Materials and Methods

### 2.1. Reagents and Strain

The fungus *P. roridum* VKM F-3565 was obtained from the all-Russian collection of microorganisms (VKM) of IBPM RAS.

2,2′-Azino-*bis*(3-ethylbenzothiazoline-6-sulfonic acid) (ABTS) was obtained from AppliChem GmbH (Darmstadt, Germany); syringaldazine (4-hydroxy-3,5-dimethoxybenzalehyde) and SigmaMarker High Range (36–205 kDa) were from Sigma-Aldrich (St. Louis, MO, USA); 2,6-dimethoxyphenol, *trans*-ferulic acid, and coniferyl alcohol were from Sigma-Aldrich (USA); Endo H_f_ Kit and DpnI were from NEB (Ipswich, MA, USA); pET-22b(+) and GeneJET Plasmid Miniprep Kit were from Thermo Scientific (Vilnius, Lithuania); Prestained Protein Marker II (10–200 kDa) was from Servicebio (Wuhan, China); and HF-Fuzz was from Dialat (Moscow, Russia). All other chemicals, used in this work, were of analytical grade and purchased locally.

### 2.2. Plasmid Construction and Laccase Gene Expression

#### 2.2.1. Construction of an Expression Vector and Heterologous Expression of Recombinant RecLacF-3565 Laccase in *E. coli* Cells

The original pET-22b(+) plasmid was linearized by PCR amplification using the pET-TEDA-F (ATGTATATCTCCTTCTTAAAGTTAAACAA) and pET-TEDA-R (TGAGATCCGGCTGCTAACAA) primers. The obtained PCR product was treated with DpnI (NEB, Ipswich, MA, USA) and purified using a Cleanup Standard kit (Eurogen, Moscow, Russia). The complete sequence of a laccase gene from the fungus *P. roridum* VKM F-3565 without the signal sequence was obtained by PCR amplification, using the F3565-pET-TEDA-F (GAAGGAGATATACATATGATTTTCCATTCCCTTTTGACGA) and F3565-pET-TEDA-R (AGCAGCCGGATCTCATTAAATTCCCGAATCTTCTTGCTCC) primers and the previously obtained cDNA as a template [41].

The expression vector pET22b-F3565 was obtained by the T5 exonuclease-dependent assembly (TEDA) cloning method [50] and was transformed into chemically competent cells of *E. coli* DH5α by the standard heat shock method [51].

Plasmid DNA was isolated using the GeneJET Plasmid Miniprep Kit (Thermo Scientific, Vilnius, Lithuania) according to the manufacturer’s recommendations. The presence of the insert and the open reading frame were verified by sequencing, using the commercial service Eurogen (Moscow, Russia).

The obtained expression vector pET22b-F3565 was transformed into the chemically competent *E. coli* BL21 (DE3) cells using heat shock method [51]. The induction of the target protein expression was performed when the optical density of the cell suspension reached 0.6 at 600 nm after adding of 0.5 or 1 mM isopropyl-β-D-1-thiogalactopyranoside (IPTG). In some cases, CuSO_4_ was also added to a final concentration of 0.25 mM. The cells were incubated at 30 °C at 200 rpm for 3 h and pelleted at 4000 g for 10 min at 4 °C. Then, the cells were disrupted by ultrasound (40% amplitude, 4.5 min of process time, 30 s of impulse time, 1 min of cool-down time (Q700 sonicator, Qsonica, Newtown, CT, USA), centrifuged at 5000× *g* and 4 °C for 10 min, and the supernatant was collected for subsequent study.

The laccase activity was determined by the rate of ABTS oxidation at 436 nm as well as the expression efficiency, and the molecular mass of the recombinant laccase were determined by SDS-electrophoresis as described below.

#### 2.2.2. Construction of an Expression Vector and Heterologous Expression of the Recombinant RecLacF-3565 Laccase in *K. phaffii* Cells

The original plasmid pPIC9K (Invitrogen, Carlsbad, CA, USA) was linearized by PCR with primers pPIC-TEDA-F (TAATTCGCCTTAGACATGACTGTTCCTCAG) and pPIC-TEDA-R (AGCTTCAGCCTCTCTTTTCTCGAGAGATAC).

The complete nucleotide sequence of the spliced gene of the alkaliphilic laccase of the fungus *P. roridum* VKM F-3565 was amplified from the previously obtained cDNA [41], using primers F3565-pPIC-F (AGAGAGGCTGAAGCTGCCTCGGTTTCACCTAATTCG) and F3565-pPIC-R (GTCTAAGGCGAATTAAATTCCCGAATCTTCTTGCTCC).

The expression vector pPIC9K-F-3565 was obtained by the T5 exonuclease-dependent assembly (TEDA) cloning method [50], linearized with SacI restriction enzyme, and transformed into the yeast cells of *K. phaffii* GS115 by electroporation according to the standard protocol (Pichia Expression Kit, Invitrogen). Electroporation was performed using a MicroPulser™ instrument (Bio-Rad, Hercules, CA, USA) at 2.0 kV for 5 ms. Then, the cells were seeded on MD agar plates (Pichia Expression Kit, Invitrogen, Carlsbad, CA, USA) and cultivated for five days at 30 °C.

A primary selection of transformants was carried out on the selective agar medium MM (Pichia Expression Kit, Invitrogen, Carlsbad, CA, USA) without the addition of histidine at 30 °C for five days.

In addition, the ability of the selected transformants to maintain growth on YPD agar medium (Pichia Expression Kit, Invitrogen, Carlsbad, CA, USA) supplemented with 2 mg/mL geneticin at 30 °C was assessed. The original cells of *K. phaffii* GS115 strain were used as a negative control.

Selection of transformants with the highest laccase production was performed by assessing the intensity of green halo formation around colonies during growth on MM medium supplemented with 0.2 mM CuSO_4_, 0.2 mM ABTS, and 2% methanol at 30 °C for 5 days. The cells of *K. phaffii* GS115 strain carrying the pPIC9K expression vector were used as negative controls.

The selected transformant was cultured until OD_600_ = 5.0 in liquid BMGY medium (Pichia Expression Kit, Invitrogen, Carlsbad, CA, USA) at 30 °C with agitation at 200 rpm. Then the cells were transferred to 500 mL of BMMY medium (Pichia Expression Kit, Invitrogen, Carlsbad, CA, USA) with the addition of 0.2 mM copper sulfate and were cultivated for seven days at 30 °C and 200 rpm. Methanol as an inducer was added every 24 h to a final concentration of 0.5%. At the peak of laccase activity, the cells were centrifuged at 5000× *g* for 10 min at 4 °C, and the obtained supernatant was used for purification of the recombinant laccase.

The production of the recombinant laccase was assessed by measuring the laccase activity in the culture medium. The laccase activity was determined by the rate of ABTS oxidation at 436 nm, considering the molar extinction coefficient ε_436_ = 29,300 M^−1^cm^−1^ [11,52].

### 2.3. Purification of the Recombinant RecLacF-3565 Laccase

The resulting supernatant (1 L) was applied to a DEAE-Toyopearl column with a carrier volume of 180 mL, equilibrated with 20 mM Na-acetate buffer, pH 5.0 (buffer A). The column was washed with 180 mL of buffer A. Laccase was eluted with a linear gradient of 0–0.5 M NaCl in the buffer A (1800 mL) at a rate of 2.0 mL/min. The fraction volume was 10 mL. Fractions with the activity toward ABTS were collected, concentrated, and desalted in an ultrafiltration cell (50 mL, Amicon, Millipore Corporation, Billerica, MA, USA) with a Millipore membrane (10 kDa). The obtained enzyme solution was applied to a Q-Sepharose column (40 mL), pre-equilibrated with buffer A. The column was washed with one volume of the same buffer. Elution was carried out with a linear gradient of 0–0.5 M NaCl in the buffer A at a rate of 2.0 mL/min. The total elution volume was 200 mL. The fraction volume was 1.1 mL. Fractions with laccase activity were pooled, concentrated, and desalted in an Amicon cell (50 mL) with a Millipore membrane (10 kDa). The resulting 6 mL sample was applied to a Resource Q column (6 mL), washed with one volume of buffer A and eluted with a linear gradient of 0–1.0 M NaCl in 60 mL of the buffer A. Fractions with peak laccase activity were combined, concentrated, and desalted in an Amicon cell (50 mL) with Millipore membrane (10 kDa). Concentration of the purified recombinant laccase was estimated by the Bradford assay [53].

### 2.4. Laccase Activity Assay

The laccase activity was determined spectrophotometrically at 436 nm by the rate of ABTS oxidation, taking into account the molar extinction coefficient ε_436_ = 29,300 M^−1^cm^−1^ [52] using a UV-160 spectrophotometer (Shimadzu, Kyoto, Japan) in a quartz cuvette (1 mL of total volume) with an optical length path 10 mm at 25 °C. The reaction mixture contained 1 mM ABTS, 20 mM Na-acetate buffer (pH 5.0) and an enzyme. One unit of the laccase activity was defined as the amount of the enzyme required to oxidize 1 μmol substrate per minute under the described conditions.

### 2.5. Recombinant Laccase Characterization

#### 2.5.1. Determination of Enzyme Molecular Mass

The apparent molecular mass of the denatured recombinant laccase was determined using SDS-electrophoresis in a 7% or 8% polyacrylamide gel according to the modified Laemmli method [54]. Proteins of Prestained Protein Marker II Kit (10–200 kDa) (Servicebio, Wuhan, China) or SigmaMarker High Range (36–205 kDa) (Sigma-Aldrich, St. Louis, MO, USA) were used as molecular mass markers. The gel was stained with Coomassie Brilliant Blue G-250 as described earlier [55]. A total of 10 µL of each experimental sample was loaded into a gel well contained 1 mg/mL total protein.

#### 2.5.2. Enzyme Deglycosilation Assay and Molecular Modelling

Deglycosylation of the recombinant laccase was carried out in denaturing and non-denaturing conditions using the Endo H_f_ kit (NEB, Ipswich, MA, USA) according to the manufacturer’s instructions (https://www.neb.com/en/protocols/2012/10/18/endo-hf-protocol, accessed on 20 January 2025; https://www.neb.com/en/protocols/2018/04/05/protocol-for-endo-h-p0702-and-p0703, accessed on 20 January 2025). About 2 µL of Endo H_f_ was used per 20 µL of the total reaction mixture. The final concentration of the recombinant laccase from *P. roridum* VKM F-3565 in all samples was 0.95 mg/mL. The residual activity of the enzyme was simultaneously tested under denaturing and non-denaturing conditions, as well as in the presence and the absence of Endo H_f_. Both the native and deglycosylated enzyme samples were then analyzed by SDS-electrophoresis in a 7% polyacrylamide gel as described above.

Identification of potential N-glycosylation sites as well as a homology modeling of the laccase of *P. roridum* VKM F-3565 with 3D-structure visualization and electrostatic potential calculations were carried out as described earlier [41].

#### 2.5.3. Determination of pH and Temperature Optima of Enzyme

The pH optimum of the purified recombinant laccase was measured in Britton-Robinson universal buffer [56] in the range of pH 2.0–9.0. 0.1 mM ABTS, 2,6-dimethoxyphenol, ferulic acid, and coniferyl alcohol, as well as 0.01 mM syringaldazine were used as substrates. The laccase activity was measured at 25 °C spectrophotometrically, as described previously, taking into account the molar extinction coefficients of the used substrates or the formed products [11].

The temperature optimum of the recombinant laccase was measured spectrophotometrically, as described above, in 20 mM Na-acetate buffer (pH 5.0) or 50 mM Tris-HCl buffer (pH 7.2) in the range of 15–65 °C, using a temperature controller from Shimadzu (Kyoto, Japan). A total of 0.1 mM ABTS was used as a substrate in the reaction.

The reaction was started by adding the laccase to the reaction mixture until a final total concentration of 0.945 μg/mL was achieved. All measurements were carried out in triplicate. The average value of maximum activity at the optimal pH or temperature was taken as 100%. The remaining values were expressed as a percentage relative to the maximum activity. All data were obtained with an error of <10%.

#### 2.5.4. Enzyme Stability Determination

Stability of the recombinant laccase in concentration of 0.15 mg/mL was analyzed under acidic conditions (in 20 mM Na-acetate buffer, pH 5.0) or neutral environmental conditions (in 50 mM Tris-HCl buffer, pH 7.2) at both 25 °C and 4 °C. The procedure of preparing enzyme samples was previously described [11]. The residual activity was measured spectrophotometrically as described above. For this, 5 μL of the incubated enzyme were added to buffer A (pH 5.0) with 0.1 mM ABTS in cuvette at 25 °C. At least three samples for each condition were used. All data were obtained with an error of <10%.

#### 2.5.5. Determination of Enzyme Activity in the Presence of Inorganic Metal Salts, Ionic and Nonionic Detergents, Chelators and Organic Solvents

To test the effect of medium components on the activity of the recombinant laccase, a reaction mixture, containing 20 mM Na-acetate buffer (pH 5.0), 0.1 mM ABTS, 0.945 μg/mL of the recombinant protein, and the test compound at different concentrations, was used. The reaction was started by addition of the laccase into the reaction mixture. The laccase activity measurements were performed at 25 °C in triplicate as described above. The average value of the enzyme activity without test compounds was taken as 100%.

Homology modeling, electrostatic potential calculation, and visualization of the results were performed as described previously [41].

#### 2.5.6. The Kinetic Data Analysis

Apparent *K*_m_ and *V*_max_ values were calculated by nonlinear least squares regression, fitting experimental data to the Michaelis Menten equation. In the case of a deviation from Michaelis-Menten equation, the data were linearized by the Lineweaver–Burk plots (1/V vs. 1/[S]) [57]. ABTS, 2,6-dimethoxyphenol (2,6-DMF), syringaldazine, ferulic acid, and coniferyl alcohol were used as substrates. The activity of the recombinant laccase was determined spectrophotometrically using 20 mM Na-acetate buffer (pH 5.0) in the reaction with ABTS, and 50 mM Tris-HCl buffer (pH 7.2) in the reaction with all other substrates. The specific activity of the enzyme was calculated considering the values of the corresponding molar extinction coefficients [11]. For each substrate concentration, the enzyme activity was determined three times. All kinetic constants were obtained with an error of <5%. The SigmaPlot program was used to process the obtained results.

### 2.6. Transformation of Phenylpropanoid and Lignin with the Recombinant Laccase

#### 2.6.1. Analysis and Identification of Phenylpropanoid Transformation Products

The transformation of phenylpropanoids was carried out in a reaction mixture, containing 2 mM aromatic substrate (ferulic acid or coniferyl alcohol), the recombinant laccase (with a final activity 4 U/mL in the reaction with ABTS), 50 mM Tris-HCl buffer with pH 7.2. The reaction was started by addition of the enzyme and incubated for 2 h at 29 °C. The reaction was stopped by addition of ethyl acetate to the reaction mixture (up to 40% of ethyl acetate, *v*/*v*). Neutral and acidic extracts of the reaction mixture were obtained in two stages [11] and used for further work. Thin layer chromatography (TLC) of the evaporated extracts on 60 F_254_ silica gel plates (Merck, Darmstadt, Germany) was applied to identify and isolate transformation intermediates [11]. The purified intermediates were analyzed using a low-resolution quadrupole mass spectrometer LCQ Advantage MAX (Thermo Finnigan, Somerset, NJ, USA) as described previously [11] and were also used for the HPLC-analysis.

The HPLC-analysis was applied to study the dynamics of phenylpropanoid loss and product formation. The reaction mixture included a total volume of 0.5 mL contained 0.1 or 1 mM phenylpropanoid, 50 mM Tris-HCl buffer (pH 7.2), and the recombinant laccase with a final activity 8 U/mL (0.0475 mg/mL) in the reaction with 0.1 mM ABTS. The reaction was initiated by addition of the enzyme into the reaction mixture. The reaction mixtures were incubated at 29 °C and 200 rpm. The experimental samples of 20 µL were taken from the reaction mixtures after 10 min, 30 min, 1 h, 2 h, 3 h, and 4 h, mixed with an equal volume of acetonitrile, and pelleted by centrifugation at 5000× *g*. The obtained supernatants were applied for subsequent analysis. At least three samples for each condition were used. A reverse-phase Phenomenex C_18_ Synergy 4u Polar-RP column (150 × 3.00 mm) was used on the HPLC system, equipped by Waters 515 HPLC pumps, Waters 2487 Dual λ Absorbance Detector (at 280 nm and 320 nm), and Waters Temperature Control Module. Elution of the phenylpropanoid oxidation intermediates was performed in isocratic concentration of acetonitrile (28% in the case of ferulic acid transformation or 30% in the case of coniferyl alcohol transformation) with 0.1% formic acid in deionized water (*v*/*v*/*v*) for 0–10 min with a rate 0.5 mL/min. The column temperature was 35 °C, and the injection volume was 20 μL.

#### 2.6.2. Analysis of Lignin Transformation Products

Samples of the reaction mixture, containing 10 mg/mL of preliminary pre-washed with deionized water dried birch lignin, 50 mM Tris-HCl (pH 7.2), and 0.0475 mg/mL of the recombinant laccase, were incubated at 29 °C (200 rpm) and collected after 24 h, pelleted at 5000× *g*, and the obtained supernatants were analyzed by HPLC. At least three samples for each condition were used. A reverse-phase Phenomenex C18 Synergy 4u Polar-RP column (150 × 3.00 mm) was used on the HPLC system equipped by Waters 515 HPLC pumps, Waters 2487 Dual λ Absorbance Detector (at 280 nm and 480 nm), and Waters Temperature Control Module. Elution of the lignin oxidation intermediates was performed using an isocratic concentration of 30% acetonitrile in deionized water (*v*/*v*) from 0 to 10 min, followed by a linear gradient from 30% to 100% acetonitrile from 10 to 14 min at a flow rate of 0.5 mL/min. The column temperature was 35 °C, and the injection volume was 20 μL. After 24 h of incubation the experimental samples were centrifuged at 5000× *g*, and the obtained pellets were dried at 100 °C until the change in mass was stopped. The obtained samples were weighed.

## 3. Results and Discussion

### 3.1. Production of the Recombinant Alkaliphilic Laccase from the Fungus of P. roridum VKM F-3565 in the E. coli Cells

**Figure 1:**
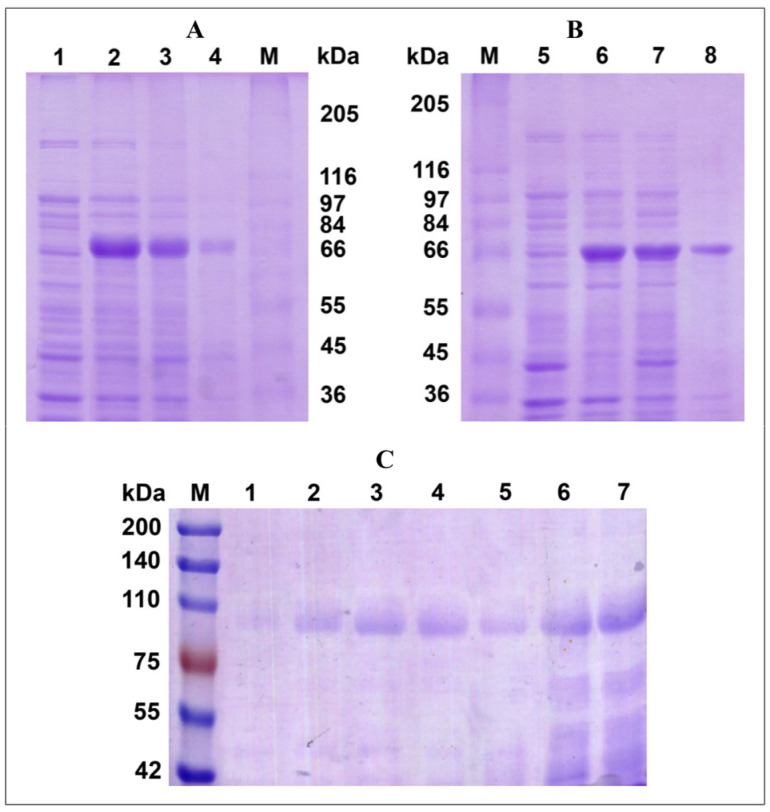
The electrophoretic analysis of heterologous expression of the recombinant laccases from *P. roridum* VKM F-3565 in *E. coli* BL21(DE3) cells ((**A**,**B**) 8% SDS-PAGE gel) and *K. phaffii* GS115 ((**C**) 7% SDS-PAGE gel) cells. (**A**,**B**) Crude extract of *E. coli* BL21(DE3) cells (induction by 1 mM IPTG): (1) before induction, (2) after induction (total protein), (3) soluble protein fraction after induction, (4) insoluble protein fraction after induction, (M) marker proteins (kDa), (5) before induction in the presence of additional 0.25 mM CuSO_4_, (6) after induction in the presence of 0.25 mM CuSO_4_ (total protein), (7) soluble protein fraction after induction in the presence of 0.25 mM CuSO_4_, and (8) insoluble protein fraction after induction in the presence of 0.25 mM CuSO_4_. (**C**) The culture liquid of *K. phaffii* GS115 cells during heterologous expression: (M) marker proteins (kDa), (1–7) days of cultivation (addition of methanol at final concentration of 0.5% each day).

Several studies have described successful expression of functional eukaryotic fungal laccases in a prokaryotic expression system based on *E. coli* (Table 1). Therefore, an attempt was made to obtain the active form of the laccase from the fungus *P. roridum* VKM F-3565 in the *E. coli* cells. For this purpose, cDNA of the laccase gene without the signal peptide was integrated into the expression vector pET-22b(+). The resulting recombinant plasmid pET22b-F3565 (Figure S1) was transformed into *E. coli* BL21 (DE3) cells. SDS-PAGE showed that upon induction of expression with 1 mM IPTG and cultivation at 30 °C, most of the recombinant protein was in soluble form (Figure 1A). The subunit mass of the recombinant laccase was about 68 kDa, which is typical for a number of fungal laccases (Table 1) [1]. However, the obtained recombinant laccase did not exhibit enzymatic activity.

It is known that the presence of Cu^2+^ ions plays an important role in the expression of the active form of laccase in both prokaryotic and eukaryotic expression systems [58]. For example, the lack of activity of the recombinant fungal laccase LacSM expressed in *E. coli* cells has been shown in the cultivation medium without CuSO_4_ [43]. Therefore, we expressed the laccase of the fungus *P. roridum* VKM F-3565 in *E. coli* BL21 (DE3) cells with the addition of 0.25 mM CuSO_4_ to the cultivation medium. The SDS-PAGE analysis showed that the recombinant enzyme was produced mainly in soluble form (Figure 1B), but the laccase activity was still absent. It is known that fungal laccases are usually glycoproteins with a carbohydrate content of 5–30% or more [59,60]. It can be assumed that the lack of activity of the obtained recombinant laccase is associated with the impaired post-translational modifications, such as glycosylation or the formation of disulfide bonds, in the prokaryotic expression system based on *E. coli*.

### 3.2. Production of the Recombinant Alkaliphilic Laccase from the Fungus of P. roridum VKM F-3565 Strain in the Yeast Cells of K. phaffii

There are numerous data describing successful production of active fungal laccases in eukaryotic, in particular yeast, expression systems (Table 1). Therefore, the laccase of the fungus *P. roridum* VKM F-3565 was expressed in an active form in the yeast cells of *K. phaffii*. For this purpose, the cDNA of the laccase gene without the signal peptide was cloned into the pPIC9K vector to obtain the expression plasmid pPIC9K-F-3565 (Figure S2), including the methanol-inducible AOX1 promoter and the recombinant laccase gene with the α-factor preproleader responsible for the laccase secretion into the culture medium. This plasmid was linearized and transformed into *K. phaffii* GS115 cells by electroporation. Colony screening with geneticin allowed the selection of colonies containing multiple copies of the laccase gene. Selection of the transformant with the maximum laccase activity was performed on the MM agar plates that contained CuSO_4_ and ABTS by screening the formation of a dark green zone around the colony. After seven days of submerged cultivation in the presence of methanol as an inducer, the yield of the recombinant laccase from *P. roridum* VKM F-3565 (RecLacF-3565) was about 20 ± 1.5 mg/L with a specific enzyme activity of about 154.0 U/mg in the reaction with 1 mM ABTS. Thus, the obtained yield of the recombinant laccase was comparable with the best yields of known recombinant laccases (Table S1).

### 3.3. Role of Glycosylation in the Recombinant Laccase Functioning

**Figure 2:**
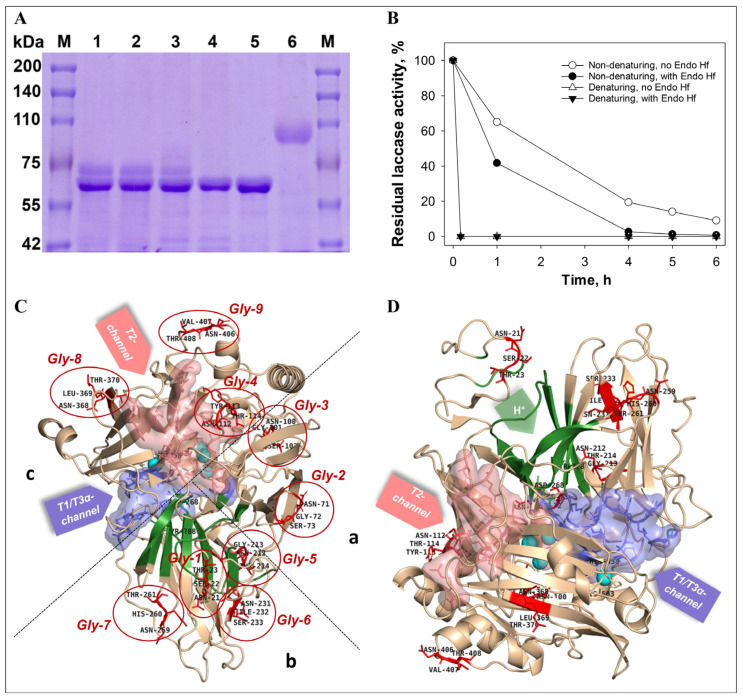
Influence of N-deglycosylation on the molecular mass (**A**) and the activity (**B**) of the recombinant laccase from *P. roridum* VKM F-3565 (the conditions were used according to the manufacturer’s instruction for Endo H_f_ Kit (NEB)) as well as distribution of the predicted N-glycosylation sites on the surface of the laccase molecule of *P. roridum* VKM F-3565. (**C**) Top view. (**D**) Side view. A (SDS-PAGE electrophoresis, 7% gel): (M) marker proteins, (1) denaturing conditions after 1 h of incubation, (2) denaturing conditions after 6 h of incubation, (3) non-denaturing conditions after 1 h of incubation, (4) non-denaturing conditions after 6 h of incubation, (5) endoglycosidase H_f_, and (6) the recombinant laccase from *P. roridum* VKM F-3565. The laccase domains are marked as a, b, and c.

According to the results of the SDS-electrophoresis, the molecular mass of the RecLacF-3565 was about 97 kDa (Figure 1C and Figure 2A), what is 10 kDa higher than the mass of the native laccase from *P. roridum* VKM F-3565 (WtLacF-3565), which has a molecular weight of 80 kDa and a mass of deglycosylated form of 67.6 kDa [41]. The difference between the molecular masses of the recombinant and wild type enzymes is most likely due to increased glycosylation of the protein in yeast cells of *K. phaffii*.

As a result of deglycosylation of RecLacF-3565 by Endo H_f_, which cleaves within the chitobiose core of high mannose and some hybrid oligosaccharides from N-linked glycoproteins, the recombinant laccase lost activity under both denaturing (fast) and non-denaturing conditions (slow) (Figure 2B). The results of the SDS-PAGE electrophoresis of the recombinant laccase samples, incubated in denaturing conditions, showed the absence of the native laccase band (97 kDa) and the appearance of the lower band (about 67–68 kDa) both after 1 h and 6 h of incubation with Endo H_f_, displaying the complete deglycosylation of the enzyme after 1 h treatment (Figure 2A). The appearance of the intermediate band with mass of about 80 kDa as well as the band with mass of 67–68 kDa were observed after 1 h incubation under non-denaturing conditions (Figure 2A). The subsequent incubation of the enzyme with Endo H_f_, under non-denaturing conditions for 6 h, resulted in precipitation of the recombinant laccase and disappearance of the corresponding deglycosylated band of 67–68 kDa in the gel, with concomitant full disappearance of the laccase activity (Figure 2A,B). The mass of the deglycosylated recombinant laccase obtained in the *K. phaffii* cells (67–68 kDa) corresponded to the mass of the inactive soluble recombinant laccase obtained in *E. coli* cells (about 68 kDa).

Previously, the presence of 9 N-glycosylation sites for the *P. roridum* VKM F-3565 laccase based on the primary sequence of the enzyme was predicted [41]. The structural analysis of the spatial localization of N-glycosylation sites in the laccase (Gly-1-Gly-9) showed the presence of four sites in domain b, three sites in domain a and two sites in domain c (Figure 2C,D). There were no N-glycosylation sites on the T1-center side. However, Gly-2, Gly-3, Gly-4, Gly-8, and Gly-9 sites surrounded the T2/T3 center as well as Gly-1, Gly-5, Gly-6, and Gly-7 were located around the laccase proton channel. The last two glycosylation regions mentioned above are in functionally significant regions of the laccase molecule. All N-glycosylation sites, except for the slightly submerged Gly-3 site (Asn100-Gly101-Ser102), were exposed on the surface of the molecule. In our experiment, deglycosylation of surface N-glycosides was accompanied by a complete loss of the recombinant enzyme activity. It is possible that the lack of any glycosylation in the prokaryotic *E. coli* expression system does not allow the production of an active recombinant enzyme.

### 3.4. Kinetic Properties of the Recombinant Laccase

The recombinant laccase from *P. roridum* VKM F-3565 was the most specific for syringaldazine (*k*_cat_/*K*_m_ = 27.0 min^−1^ µM^−1^) (Table 2). Among phenylpropanoids, ferulic acid was the best substrate (*k*_cat_/*K*_m_ = 2.2 min^−1^ µM^−1^). The laccase RecLacF-3565 increased the rate of ABTS oxidation by six times compared to WtLacF-3565. At the same time, a decrease in the values of catalytic constants of RecLacF-3565 was noted in the oxidation reactions of other phenolic compounds and phenylpropanoids with a corresponding decrease in specificity.

### 3.5. Temperature and pH Optima, Stability of the Recombinant Laccase

**Figure 3:**
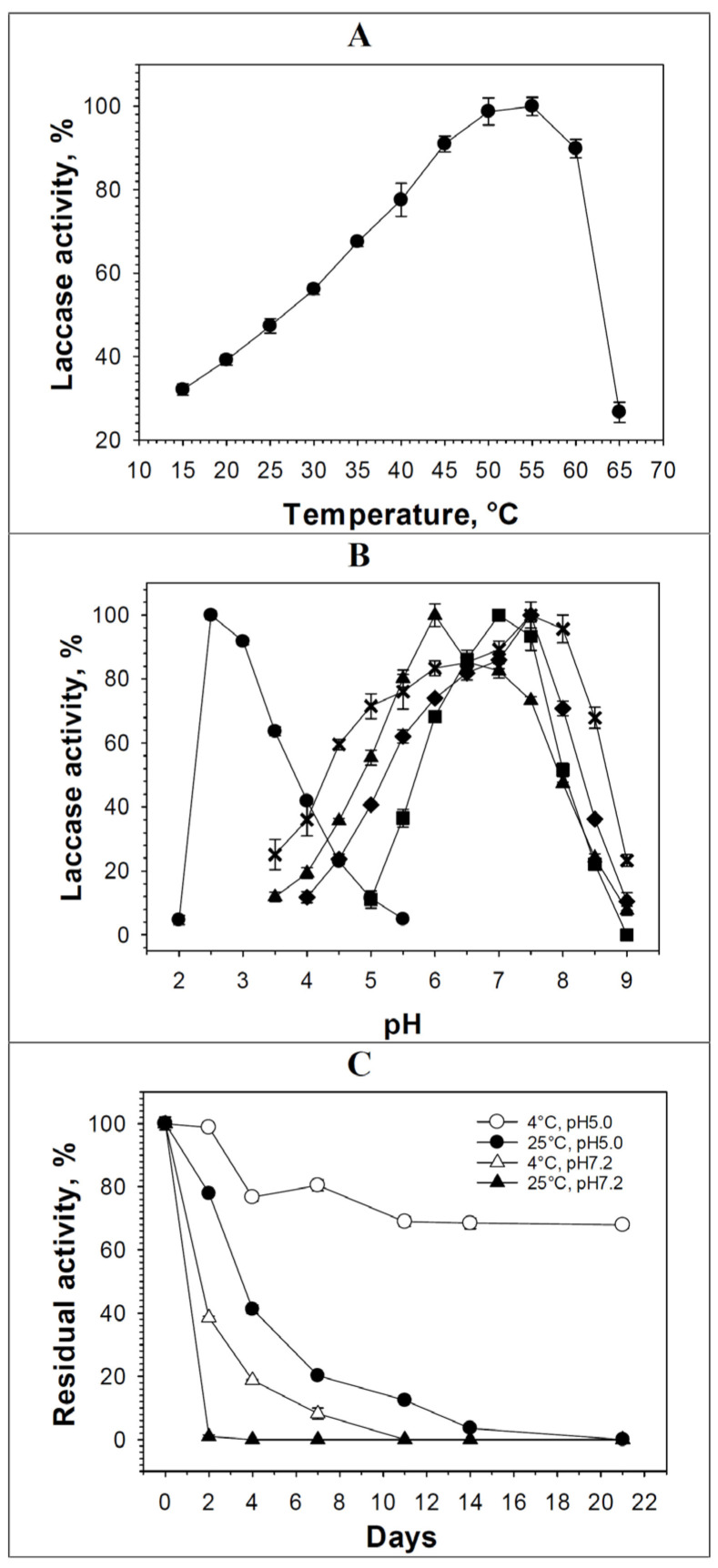
Properties of the recombinant laccase from *P. roridum* VKM F-3565: (**A**) temperature optimum; (**B**) pH optimum in the reaction with different substrates (ABTS (circle symbols), syringaldazine (triangle), 2,6-dimethoxyphenol (square), coniferyl alcohol (diamond), and ferulic acid (x)); and (**C**) stability of the enzyme at different temperatures and pH.

The recombinant laccase from *P. roridum* VKM F-3565, obtained in *K. phaffii* cells, oxidized ABTS in the range of 15–65 °C with maximum activity at 55 °C (Figure 3A), which is 10 °C lower than the temperature optimum of WtLacF-3565 [41]. The known recombinant alkaliphilic fungal laccases also have a T optimum in the range of 45–70 °C (Table 1).

Like the laccase WtLacF-3565 [41], the laccase RecLacF-3565 exhibited maximum activity against the tested phenolic compounds and phenylpropanoids in a neutral environment (pH 6.0–7.5) (Figure 3B, Table 1). However, the pH optimum of the oxidation of syringaldazine and 2,6-dimethoxyphenol by the recombinant laccase RecLacF-3565 was slightly more acidic than that of WtLacF-3565, while phenylpropanoids were oxidized by RecLacF-3565 with maximum activity at higher pH values than by WtLacF-3565 (Figure 3B, Table 1) [41]. The pH optimum of ABTS oxidation by laccases RecLacF-3565 and WtLacF-3565 was in the acidic pH region with a small difference (pH 2.5 and pH 2.8, respectively), as in many known fungal laccases, what is associated with the nature of the substrate itself [1].

The recombinant laccase from *P. roridum* VKM F-3565 was most stable during storage at 4 °C and pH 5.0 (70% of the residual activity was observed after 21 days of incubation under these conditions) (Figure 3C). The residual activity of RecLacF-3565 samples was about 77% after two days and 42% after four days of incubation at 25 °C and pH 5.0 (Figure 3C). 38% of the residual activity was observed after four days of an incubation under neutral conditions (pH 7.2) at 4 °C and 18% of the residual activity was after two days of incubation under the same conditions (Figure 3C). As a result of incubation of the recombinant laccase samples at 25 °C in a neutral medium, about 3% of the residual laccase activity was observed after two days.

The enzyme could withstand freezing at −70 °C in 20 mM Na-acetate buffer with pH 5.0 without losing its activity. When the enzyme was thawed after storage at −20 °C in 20 mM Na-acetate buffer with pH 5.0, the laccase retained 92.2% of the initial activity.

### 3.6. The Influence of Metal Salts, Ionic and Nonionic Detergents, Cation Chelators and Organic Solvents on the RecLacF-3565 Laccase Activity

Chlorides of all metals studied at a concentration of 100 mM inhibited the recombinant laccase both under acid and neutral conditions (Table 3). On the contrary, similar sulfates and phosphates activated the recombinant enzyme at pH 5.0. Copper, manganese, and zinc sulfates increased the enzyme activity by 2.9, 2.5 and 2.7 times, respectively. In a neutral environment, most sulfates had a barely noticeable inhibitory effect, except for zinc sulfate, which reduced the activity of the recombinant laccase by more than three times. Note that Cu^2+^ ions caused autooxidation of the 2,6-dimethoxyphenol substrate in a neutral medium (50 mM Tris-HCl, pH 7.2), and therefore, their effect on recombinant laccase was not studied. Overall, the recombinant laccase was more tolerant to monovalent metal chlorides and ammonium chloride in a neutral environment than in an acidic environment.

In the presence of nonionic surfactants at a micelle-forming concentration of 1%, the activity of the recombinant laccase decreased to 20–52% of the control at pH 5.0 (Table 4). Tween 40 had the smallest inhibition, and Triton X100 turned out to be the most powerful inhibitor. The inhibitory effect of the nonionic surfactants was significantly reduced in a neutral environment. The ionic detergent SDS at concentrations of 1–2% slightly activated the recombinant laccase at pH 5.0 (106–116% of the activity in the control), while at neutral pH it had an inhibitory effect (Table 4). Another ionic detergent, the CTAB, in the tested concentration range of 0.25–2%, almost completely inhibited the recombinant laccase in both acidic and neutral environments (Table 4).

The recombinant laccase was completely inhibited in the presence of 0.5% or higher of EDTA (chelating agent) at pH 5.0, while at pH 7.2 EDTA had an activating effect in all tested concentrations (Table 4). It is possible that the lack of chelating effect of EDTA under neutral conditions is a result of the acquisition of a negative charge by the amino acid environment of the T1-site (Figure S3) and the EDTA molecule, which prevents the entrance of EDTA into the cavity of the T1-center.

In acidic environment, the most tested organic solvents at a concentration of 20% completely inhibited RecLacF-3565, except for ethanol, in the presence of which the enzyme retained 22% activity (Table 4). In the presence of 10% organic solvents in the reaction mixture, the activity of RecLacF-3565 decreased to 9.4–52% depending on the used solvent (Table 4). The smallest inhibitory effect was observed with ethanol, the highest inhibition was in the presence of DMSO. In a neutral environment, the recombinant laccase showed slightly greater tolerance in relation to the organic solvents, although their inhibitory effect was retained.

According to our calculations of the distribution of electrostatic potential on the Connolly surface of the RecLacF-3565 (Figure S3), the surface of the laccase molecule, including the surface of the cavity of the T1-active center, acquire an increased negative charge under an increasing the pH of the solution. It most likely affects T1-center interaction with various salt ions, as well as with other tested effector molecules. The probability of entering the cavity of the substrate-binding T1-active center decreases for negative ions and, conversely, increases for positive ions at neutral pH. The same effects may also occur for the T2-channel and the proton channel environment, the electrostatic potential of whose surface also changes with changes in pH.

### 3.7. Transformation of Phenylpropanoids and Lignin by the Recombinant Laccase

**Figure 4:**
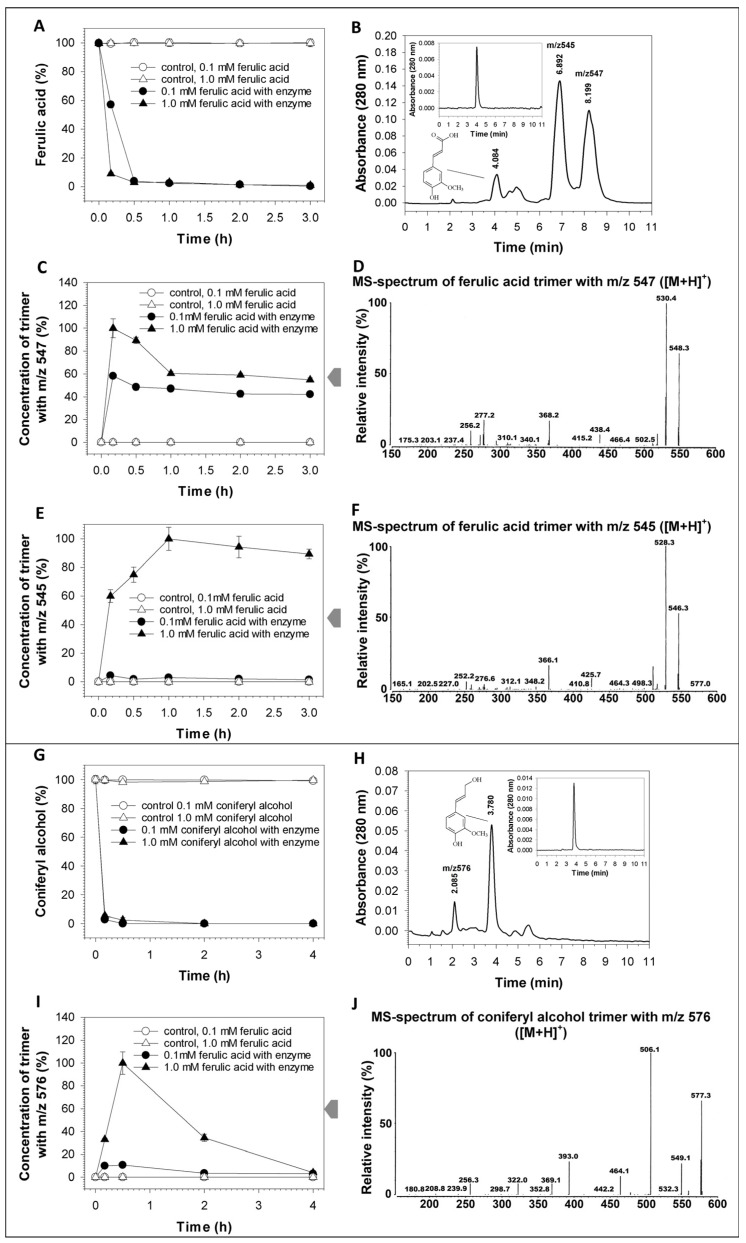
Dynamics of ferulic acid (**A**–**F**) and coniferyl alcohol (**G**–**J**) transformation and corresponding products formation catalyzed by the recombinant laccase from *P. roridum* VKM F-3565 under neutral conditions. The average values are given based on the results of at least three replicates with an error of <5%. Chromatograms of the reaction mixtures containing ferulic acid (**B**) or coniferyl alcohol (**H**) as substrates and the recombinant laccase as catalyst are shown as examples. The chromatograms of the standard compounds (10^−5^ M), used in the experiment as the initial substrates, are shown as insertions (*trans*-ferulic acid (128708-5G, Sigma-Aldrich, St. Louis, MO, USA) and coniferyl alcohol (223735-100 mg, Sigma-Aldrich, St. Louis, MO, USA)).

The recombinant laccase RecLacF-3565 almost completely removed 0.1 and 1 mM ferulic acid from the reaction mixture after 10–30 min of incubation at pH 7.2 (Figure 4A), forming two predominant trimeric products with [M+H]^+^546 and [M+H]^+^548 (Figure 4B–F, Table 5), similar to WtLacF-3565 laccase [41]. As noted earlier [41] and shown in this work (Figure 4D,F), the product with m/z547 was most likely a protonated derivative and precursor of the product with m/z545. Increased accumulation of the two intermediates was achieved by increasing the concentration of the original substrate. The maximum yields of the compounds with m/z547 and m/z545 were approximately 0.08 and 0.86 mM after 10 min and 1 h of 1 mM ferulic acid oxidation, respectively (Figure 4C,E). Subsequent incubation of the reaction mixtures led to the further slower partial oxidation of the accumulated compounds by the recombinant laccase. Thus, varying the concentration of the initial substrate and the time of the reaction mixture incubation makes it possible to regulate the yield of the target products.

Coniferyl alcohol in concentration of 0.1 and 1.0 mM was faster removed (during 10 min) from the reaction mixture by the recombinant laccase at the same environmental conditions (Figure 4G, Table 5). A predominant trimeric product with [M+H]^+^577 identical to the product of transformation of coniferyl alcohol by laccase WtLacF-3565 [41] was observed (Figure 4H–J). The maximum yield of the intermediate (approximately 0.61 mM) was achieved after 30 min of transformation of 1 mM coniferyl alcohol followed by subsequent oxidation of the intermediate by the recombinant laccase. No significant accumulation of the other oxidation products during transformation of coniferyl alcohol was detected during this time at 280 and 320 nm.

Among the known recombinant alkaliphilic laccases of fungi, only a pair of enzymes catalyzing the reaction of protein–protein cross-linking using mediators under neutral conditions are known (Table 1): laccase from *Paramyrothecium* sp. strain MM13-F2103 [42] and laccase from *Chrysocorona lucknowensis* strain 5537 [29]. There is also no information concerning oligomerization of phenylpropanoids and phenolic compounds by the known recombinant alkaliphilic fungal laccases (Table 1).

Currently, there are few studies devoted to the oligomerization of such lignin structural units as coniferyl alcohol and ferulic acid using native fungal laccases (Table 5). However, the oligomerization of coniferyl alcohol by fungal laccases has been least studied. Thus, two dimers with MW = 340 g/mol [31] as well as trimers with molecular weights of 432 and 436 [11] were reported. There is a work describing the chemical-enzymatic production of the trimer (*β*-5/*β*-O-4) of coniferyl alcohol, where fungal laccase is used to obtain the dimer as a trimer precursor [61]. Oligomers with *β*-*β*, *β*-5 and *β*-O-4/*α*-O-4 structure were also obtained using laccases from *Trametes versicolor* and *Rhus vernicifera*, and it was shown that compounds with a branched *β*-O-4/*α*-O-4 structure predominate in a near-neutral environment, while in an acidic environment, oligomers with linear *β*-*β* and *β*-5 structures are predominantly formed, regardless of the laccase used [70]. Several natural oligomers of coniferyl alcohol are also known including the folloiwng: dimers with *β*-*β* (MW = 358 g/mol), *β*-5 (MW = 358 g/mol), and *β*-O-4 (MW = 376 g/mol) linkage as well as trimers with *β*-5/*β*-O-4 (MW = 584 and 618 g/mol), *β*-*β*/*β*-O-4 (MW = 584 g/mol), and *β*-O-4/*β*-O-4 (MW = 572 g/mol) linkage [62]. Dimers similar to natural ones (pinoresinol (*β-β*) or dimers with *β*-5 and *β*-O-4 structure) were found during oligomerization of coniferyl alcohol by plant laccase from *Rhus vernicifera* Stokes [71]. The coniferyl alcohol trimer with MW = 576 g/mol obtained in the present work does not correspond to previously known natural trimers and trimers obtained using other fungal laccases. Thus, fungal laccases are capable of oligomerizing coniferyl alcohol to form dimers and trimers with a molecular weight different from the corresponding natural oligomers.

The fungal laccases are also able to oligomerize ferulic acid to form *β*-*β*, *β*-5, *β*-4 and *β*-O-4 dimers with molecular weights in the range of 298–403 g/mol, as well as trimers with MW = 534–636 g/mol and tetramers with MW = 735–770 g/mol with an unknown binding type (Table 5). Among the natural oligomers dimers with *β*-5 (MW = 358 g/mol) and 5-5 (MW = 344 g/mol) binding, trimers with *β*-5/*β*-O-4 (MW = 578 g/mol), *β-β*(cyclic)/*β*-O-4 (MW = 578 g/mol), 5-5/*β*-O-4 (MW = 578 and 596 g/mol) and *β*-O-4/*β*-O-4 (MW = 578 g/mol) structures are known [67,68,69]. The two trimers of ferulic acid obtained in the present work have molecular weights that are different from all currently known natural trimers and those synthesized using fungal laccases. Thus, fungal laccases most often catalyze the formation of oligomers other than natural compounds.

It was previously shown that laccases could perform both depolymerization of lignin with a release of low molecular weight products (oligo- and monomeric-compounds such as ferulic acid, aniline, acetosyringone, syringaldehyde, and acetovanillone) and its modification [72,73,74]. In most cases, these reactions are carried out by fungal laccases in an acidic environment, and the above-described types of reactions are usually studied separately from each other. Thus, it is completely unclear whether the same fungal laccase, particularly the alkaliphilic laccase, can oligomerize phenylpropanoids (lignin monomers) and simultaneously depolymerize lignin itself.

**Figure 5:**
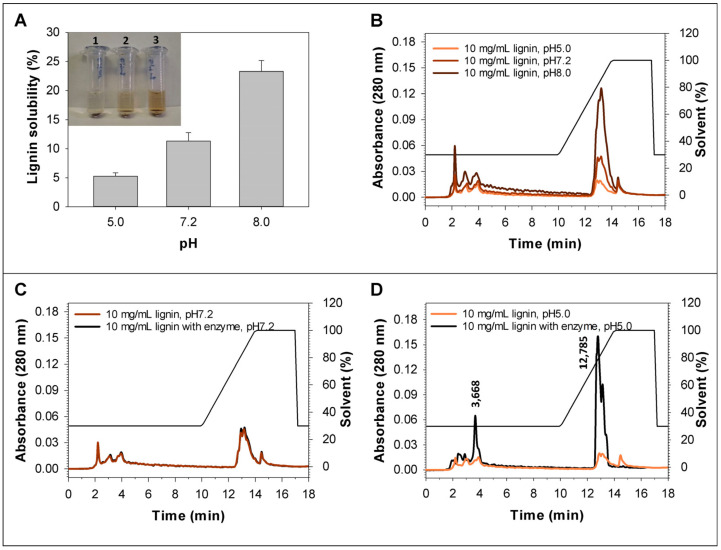
Solubility of 10 mg/mL birch lignin in buffers with different pH levels (pH 5.0 is 20 mM Na-acetic buffer, pH 7.2 and pH 8.0 are 50 mM Tris-HCl buffer) and the lignin degradation by the recombinant laccase from *P. roridum* VKM F-3565 under neutral and acid conditions. (**A**) The changes in the color of the lignin solutions after 24 h of incubation (at pH 5.0 (1), pH 7.2 (2), and pH 8.0 (3)) and the quantitative analysis of lignin solubility based on the determination of the difference in mass between the initial dry lignin and undissolved dried lignin after 24 h incubation in an appropriate buffer. (**B**) The chromatogram of the dissolved birch lignin after 24 h of incubation in buffers with different pH. (**C**,**D**) The chromatograms of the reaction mixtures of the dissolved birch lignin without and with the recombinant laccase after 24 h of incubation under neutral and acid conditions, correspondingly.

According to the previously published work, lignins can be partially dissolved in a buffer with neutral pH [75]. Considering this fact, the solubility of birch lignin in 50 mM Tris-HCl buffer with pH 7.2 was estimated, and it was shown that on average about 11.0 ± 1.3% of the lignin added to the buffer is converted into a soluble form and stains the buffer (Figure 5A,B). This corresponds to a concentration of about 1.1 g/L of the dissolved lignin, which is sufficient for the determination of the lignin degradation products in the presence of the recombinant laccase by the HPLC-analysis, if the degradation process occurs. In this work, the HPLC analysis did not reveal any noticeable changes in the chromatogram of the dissolved fraction of birch lignin incubated 24 h in the presence of the recombinant laccase RecLacF-3565 under neutral conditions, compared to the control variant in the absence of the enzyme (Figure 5C). Moreover, the insoluble lignin mass was not changed during the experiment after 24 h of incubation of the reaction mixture. However, the recombinant laccase showed activity towards the lignin under acidic conditions (pH 5.0). After 24 h of incubation, the compounds with *R*_t_ = 3.668 min and *R*_t_ = 12.785 min were accumulated in the medium (Figure 5D). Thus, considering the relatively high rate of the phenylpropanoid oligomerization and the absence of the lignin depolymerization under neutral pH conditions, it can be concluded that the recombinant laccase RecLacF-3565 is capable of catalyzing specific one-way oligomerization of phenylpropanoids in the neutral environments.

## 4. Conclusions

In this work, the possibility of the obtaining the recombinant alkaliphilic laccase from *P. roridum* VKM F-3565, using a *K. phaffii* transformant with a yield of 20 mg/L, was shown. For the first time, the one-way oligomerization of phenylpropanoids in a neutral medium was demonstrated among all known recombinant alkaliphilic fungal laccases. The advantage of the obtained recombinant enzyme is its ability to oxidize phenylpropanoids without any mediators, which significantly reduces the cost of the production process. The obtained recombinant laccase differed from the wild-type laccase in the degree of glycosylation and increased the pH optima of the phenylpropanoids oxidation reaction, which had a positive effect on the production of oligomeric products, given the facilitation of polymerization with increasing pH. The enzyme exhibits greater resistance to surfactants and the EDTA in neutral rather than acidic conditions, whereas its tolerance to mono- and divalent-metal ions is higher at acidic pH. A significant role of exclusively N-glycosylation of a molecule of the alkaliphilic laccase of *P. roridum* VKM F-3565 in its functional activity was also shown. Considering the catalysis of one-way oligomeriztion of phenylpropanoids (the natural precursors of lignins and lignans), the unique recombinant alkaliphilic laccase can be used for selective biosynthesis under neutral conditions of novel oligomeric derivatives of phenylpropanoids for industrial and pharmacological purposes.

## Figures and Tables

**Table 1:** The known fungal laccases, active mostly in a neutral or close to neutral alkaline environment. Abbreviations: ABTS—2,2′-azino-*bis*(3-ethylbenzothiazoline-6-sulfonic acid, SGZ—syringaldazine, 2,6-DMP—2,6-dimethoxyphenol.

Laccase Source	Production Host	GenBank Acc. No.	MW (SW), kDa	T-Optimum, °C	pH- Optimum	Tested Substrates	Enzyme Yield or Specific Activity	Reference
Basidiomycetes								
*Coprinus cinereus* laccase Lcc9	*Aspergillus niger* MA70.15	BK004119.1	No info	No info	6.0 7.5	SGZ dyes (M2GE, KD8B, KM8B, K7R, 6B, IC)	150 U/L 3138 ± 62 U/L	[14]
*Coprinus cinereus* (Lcc1)	*Aspergillus oryzae*	AF118267.1	(66)		6.0–7.0	SGZ	135 mg/L	[15]
*Coprinopsis cinerea* strain Okayama 7, Lcc9	*Pichia pastoris* GS115/pPIC9K	BK004119.1	60.2	60–70	6.0 (with SGZ, 70–75% activity under pH 7.0–8.0)	SGZ, dyes	1.750–3.138 U/L (315.3 U/mg)	[16,17]
*Coprinopsis cinerea* (*Coprinus cinereus*) PIE5—mutant Lcc9	*Pichia pastoris* GS115/pPIC9K	no	(61)	60	8.5 8.0 7.0	guaiacol 2,6-DMP indigo blue	318.4 U/mg (towards ABTS)	[18,19]
*Rhizoctonia solani* *wt-lcc4*	*Aspergillus oryzae*	Z54277.1	130 (66)	No info	6.0–7.0	SGZ	No info	[20]
*Rhizoctonia praticola* Lac I, Lac IIa, Lac IIb	Not recombinant	no	215 175 (78) 68	60	7.4	SGZ	2075.9 nkat/mg 1022.0 nkat/mg 692.3 nkat/mg	[21]
*Pleurotus eryngii* (PEL3)	*Aspergillus niger* MGG029strain/pPEL3G expression vectors	AY686700.1	(58)	No info	6.0	2,6-DMP	1.3 U/mg (for pPEL3G), 2.4 U/mg	[22]
*Pleurotus sajor-caju* strain P32-1, *Psc lac4* gene	*Pichia pastoris* GS115	AF297528.1	(59)	No info	6.0 6.5 7.0	2,6-DMP SGZ guaiacol	2100 U/mg (towards ABTS), 4.85 mg/L	[23]
Laccases from *Pycnoporus cinnabarinus* and basidiomycete PM1 L3, L4, L5	*Pichia pastoris*, *Saccharomyces cerevisiae*	No info	No info	No info	7.0–8.0 8.0–9.0	2,6-DMP guaiacol	No info	[24]
*Schizophyllum commune* *scL12*, *scL* genes	*Nicotiana tabacum*	AB015758.1	No info	No info	6.0	2,6-DMP, trichlorophenol	13.1 nkat/g 6.7 nkat/g	[25]
*Trametes versicolor* Lcc4, LCC5	*Pichia pastoris* GS115, expression vector pPIC9	U44431.1 Q12717.1	No info	No info	7.0	aflatoxin B1, zearalenone	No info	[26]
*Trametes versicolor* DSM 11,269 (Lcc1)	*Y. lipolytica* MY1212	X84683.1	75	50	4.0–7.0	dyes (Amaranth, Direct Blue 71, Reactive Black 5, RBBR, Acid Violet 17)	0.25 U/mL	[27]
Ascomycetes								
*Acremonium murorum* war. *murorum* CBS 157.72	*Aspergillus awamori* AWC4.20, expression vector pAWGLA2, pUR7893 *Saccharomyces cerevisiae* strain VW-K1	AJ271104.1	(67)	60	8.5–9.0	SGZ	600 mg/L	[28]
*Chrysocorona lucknowensis* strain 5537	*Escherichia coli*	PA565680.1	No info	No info	7.0	Protein–protein cross-linking (pea, soybean, and wheat proteins) using DL-catechin as mediator	No info	[29]
*Cochliobolus heterostrophus* (BD22449) *Fusarium verticillioides* (BD22865)	*Aspergillus*	No info	No info	No info	8.0	SGZ	No info	[30]
*Curvularia geniculata* VKM F-3561 oxidase I oxidase II	Not recombinant	OR250480.1	1035 (>200) 870 (>200)	70	7.0–7.5, 7.5–8.5 6.8–7.0 7.0–8.0	SGZ 2,6-DMP ferulic acid coniferyl alcohol	15.4 U/mg 22.9 U/mg	[31]
*Melanocarpus albomyces* IMI 255,989 strain	*Saccharomyces cerevisiae*	AJ571698.1	(80)	70	7.5	guaiacol	No info	[32,33,34]
*Melanocarpus albomyces*, *lac1* gene	*Saccharomyces cerevisiae*, expression vector pYES2	AJ571698.1	(100)	No info	4.0–5.0 (70% activity under pH 7.0)	2,6-DMP	4.5 nkat/mL (towards ABTS), 7.0 mg/L (102 nkat/mg)	[35]
*Melanocarpus albomyces* VTT D-96490	Not recombinant	AJ571698.1	(80)	60–70	6.0–7.0 5.0–7.5	SGZ guaiacol	836 nkat/mg (towards ABTS)	[36]
*Melanocarpus albomyces* VTT D-96490	*Trichoderma reesei* RutC-30, pLLK13	AJ571698.1	(71.3)	No info	6–7.5 5.0–7.5	SGZ, guaiacol	193 nkat/mL, 230 mg/L	[37]
*Moniliophthora perniciosa* FA553, *LacMP* gene	*Pichia pastoris* X33, expression plasmid pPICZaA-6AA-*LacMP*	ABRE01017453.1	(57)	45–60	7.5 6.5 6.5	SGZ 2,6-DMP guaiacol	232 U/L	[38]
*Myceliophthora thermophila* CBS 117.65, *lcc1* gene	*Aspergillus oryzae* strain HowB104, expression vector pMWR3, pUC18, pRaMB5	XM_003663693.1	100–140 (85)	No info	6.5	SGZ	11–19 mg/L	[39]
*Myrothecium verrucaria* 24G-4	Not recombinant	No info	(62)	70	9.0	4-aminoantipyrine and phenol polymerization	1.23 U/mg (towards 4-aminoantipyrine and phenol)	[40]
*Myrothecium roridum* VKM F-3565	Not recombinant	OP168887	80 (80)	65	7.8 7.4 6.5 7.0	SGZ 2,6-DMP ferulic acid coniferyl alcohol	93.9 U/mg	[41]
*Paramyrothecium* sp. strain MM13-F2103	*Escherichia coli*	PA564876.1	No info	No info	8.5–9.0	protein–protein cross-linking (pea, soybean, and wheat proteins) using mediators (L-DOPA and DL-catechin)	No info	[42]
*Sordaria macrospora* k-hell, *lacSM* gene	*Escherichia coli* Top10 BL21(DE3) pET-30a	NC_089376.1 (locus_tag = “SMAC4_06098”) corresponding to XP_024511624.1	(67)	55–60	6.0 7.0 5.0	guaiacol SGZ 2,6-DMP	239 U/L (towards guaiacol)	[43]

**Table 2:** Kinetic properties of the recombinant laccase from *P. roridum* VKM F-3565 in comparison with the properties of the native laccase of *P. roridum* VKM F-3565. At least three experiments were carried out to calculate each kinetic parameter. All kinetic constants were obtained with an error of <5%. The *K*_m_ values were measured using 20 mM Na-acetate buffer (pH 5.0) in reaction with ABTS as a substrate, 50 mM Tris-HCl buffer (pH 7.2) in reaction with the other substrates. RecLacF-3565 is the recombinant laccase, and WtLacF-3565* is wild type laccase [41].

Substrates	RecLacF-3565				WtLacF-3565*			
	pH_opt_	*K*_m_, µM	*k*_cat_, min^−1^	*k*_cat_/*K*_m_, min^−1^·µM^−1^	pH_opt_	*K*_m_, µM	*k*_cat_, min^−1^	*k*_cat_/*K*_m_, min^−1^·µM^−1^
ABTS	2.5	3703.0	13,851.0	3.7	2.8	1135.0	2291.6	2.0
Syringaldazine	7.0	6.0	162.0	27.0	7.8	7.5	240.0	32.0
2,6-dimethoxyphenol	6.0	203	187.2	0.9	7.4	3500.0	851.7	0.3
Ferulic acid	7.5	16.7	36.0	2.2	6.5	6.2	462.4	74.6
Coniferyl alcohol	7.5	109.0	91.0	0.8	7.0	29.4	375.9	12.8

**Table 3:** The effect of salts on the activity of the recombinant laccase *P. roridum* VKM F3565 at different pH levels. The values of standard error were presented. The increase in the negative effect is indicated by the strengthening intensity of the blue color, while the growing activating effect is shown by the increasing brightness of the orange color.

Salts	Laccase Activity, %					
	pH 5.0			pH 7.2		
	5 mM	10 mM	100 mM	5 mM	10 mM	100 mM
NaCl	105.2 ± 1.3	99.4 ± 4.8	69.7 ± 1.3	100.0 ± 0.9	96.8 ± 2.0	92.8 ± 2.8
KCl	104.3 ± 7.3	101.0 ± 2.8	74.3 ± 0.5	98.5 ± 3.3	94.4 ± 0.5	89.8 ± 3.8
NH_4_Cl	103.0 ± 2.2	101.2 ± 2.5	76.0 ± 1.2	91.5 ± 1.8	90.9 ± 0.1	87.4 ± 2.3
CuCl_2_·2H_2_O	203.6 ± 2.3	114.1 ± 1.8	67.5 ± 1.9	-	-	-
MgCl_2_·6H_2_O	130.0 ± 0.3	130.3 ± 2.7	66.2 ± 3.0	96.0 ± 1.2	91.0 ± 0.5	77.6 ± 2.4
MnCl_2_·4H_2_O	153.6 ± 0.5	152.7 ± 2.2	76.9 ± 1.7	82.8 ± 1.6	78.3 ± 0.5	52.6 ± 2.9
CaCl_2_·H_2_O	138.1 ± 2.8	143.5 ± 1.0	84.6 ± 0.1	97.6 ± 8.3	97.4 ± 0.2	80.8 ± 0.8
CoCl_2_·6H_2_O	149.3 ± 4.3	149.5 ± 1.7	70.7 ± 1.7	59.8 ± 3.2	61.1 ± 1.4	47.9 ± 3.3
Na_2_SO_4_	115.0 ± 4.0	116.2 ± 5.5	167.0 ± 6.0	97.4 ± 6.1	93.6 ± 0.3	111.4 ± 2.0
K_2_SO_4_	109.1 ± 2.8	125.1 ± 3.2	164.2 ± 3.6	96.1 ± 0.6	96.5 ± 0.2	98.8 ± 1.5
(NH_4_)_2_SO_4_	112.7 ± 2.2	125.6 ± 2.2	172.4 ± 3.3	97.6 ± 0.3	97.0 ± 1.8	96.0 ± 0.3
CuSO_4_·5H_2_O	201.1 ± 3.7	239.7 ± 8.1	288.4 ± 8.3	-	-	-
MgSO_4_·7H_2_O	144.3 ± 4.0	151.2 ± 2.3	195.0 ± 1.4	95.2 ± 2.4	95.9 ± 5.9	94.2 ± 4.2
MnSO_4_·5H_2_O	168.5 ± 7.3	188.8 ± 0.6	245.3 ± 3.3	84.2 ± 1.2	80.6 ± 1.2	78.5 ± 0.9
ZnSO_4_·7H_2_O	182.7 ± 4.8	200.3 ± 5.8	266.7 ± 8.4	28.2 ± 1.8	30.0 ± 0.1	29.0 ± 1.4
NaH_2_PO_4_·2H_2_O	111.0 ± 3.0	115.4 ± 0.9	162.9 ± 8.1	141.7 ± 1.4	155.6 ± 0.8	101.2 ± 1.8
KH_2_PO_4_	108.1 ± 0.6	113.8 ± 5.7	165.0 ± 5.5	116.1 ± 2.4	141.2 ± 1.8	113.0 ± 0.6

**Table 4:** The influence of nonionic and ionic detergents, cation chelator and organic solvents on the activity of the recombinant laccase from *P. roridum* VKM F3565 at different pH levels. The values of standard error were presented. Abbreviations: SDS (sodium dodecyl sulfate), EDTA (ethylenediaminetetraacetic acid), CTAB (cetyltrimethylammonium bromide), and DMSO (dimethyl sulfoxide). The increase in the negative effect is indicated by the strengthening intensity of the blue color, while the growing activating effect is shown by the increasing brightness of the orange color.

Compound	Laccase Activity, %					
	0.25%	0.5%	1%	2%	10%	20%
pH 5.0						
Tween 20	n.d.	59.4 ± 1.1	38.0 ± 2.1	n.d.	n.d.	n.d.
Tween 40	n.d.	64.3 ± 2.6	50.8 ± 1.5	n.d.	n.d.	n.d.
Tween 60	n.d.	65.7 ± 1.1	52.0 ± 1.8	n.d.	n.d.	n.d.
Tween 80	n.d.	59.8 ± 1.1	41.8 ± 0.4	n.d.	n.d.	n.d.
Triton X-100	n.d.	37.0 ± 3.1	20.0 ± 0.5	n.d.	n.d.	n.d.
SDS	n.d.	n.d.	105.7 ± 0.7	115.5 ± 2.5	n.d.	n.d.
EDTA	28.4 ± 1.7	0.0	0.0	0.0	n.d.	n.d.
CTAB	0.0	0.0	0.0	0.0	n.d.	n.d.
Ethanol	n.d.	n.d.	n.d.	n.d.	51.6 ± 3.1	22.0 ± 1.9
Acetonitrile	n.d.	n.d.	n.d.	n.d.	18.1 ± 1.1	0.0
Acetone	n.d.	n.d.	n.d.	n.d.	28.2 ± 1.6	0.0
DMSO	n.d.	n.d.	n.d.	n.d.	9.4 ± 0.9	0.0
pH 7.2						
Tween 20	n.d.	97.4 ± 0.5	90.7 ± 0.7	n.d.	n.d.	n.d.
Tween 40	n.d.	99.8 ± 0.2	96.1 ± 1.6	n.d.	n.d.	n.d.
Tween 60	n.d.	91.8 ± 0.9	77.7 ± 0.7	n.d.	n.d.	n.d.
Tween 80	n.d.	94.2 ± 0.9	88.6 ± 0.2	n.d.	n.d.	n.d.
Triton X-100	n.d.	96.0 ± 1.5	83.6 ± 4.0	n.d.	n.d.	n.d.
SDS	n.d.	n.d	53.2 ± 3.2	29.8 ± 1.0	n.d.	n.d.
EDTA	158.7 ± 3.8	160.8 ± 3.5	161.7 ± 2.3	179.2 ± 0.6	n.d.	n.d.
CTAB	14.2 ± 2.5	0.0	0.0	0.0	n.d.	n.d.
Ethanol	n.d.	n.d.	n.d.	n.d.	60.8 ± 3.4	40.8 ± 0.8
Acetonitrile	n.d.	n.d.	n.d.	n.d.	36.1 ± 2.4	0.0
Acetone	n.d.	n.d.	n.d.	n.d.	56.1 ± 1.4	17.1 ± 1.6
DMSO	n.d	n.d	n.d	n.d	38.8 ± 1.2	14.8 ± 0.3

**Table 5:** The known natural plant oligomers of coniferyl alcohol and ferulic acid and the known products of their oligomerization catalyzed by fungal laccases.

MW, g/mol	Mass Spectrum (Relative Intensity, %)	Linkage	Oligomericity	Catalyst (pH)	Reference
**coniferyl alcohol** (MW = 180 Da)					
**576**	[M+H]^+^577(70%), 549(25%), 506(100%), 464(15%), 393(25%)	No info	Trimer	Recombinant laccase or wild type laccase from *P. roridum* VKM F-3565 (pH 7.2)	present work, [41]
432	[M+H]^+^433(100%), 432(86%), 289(59%), 273(66%)	No info	Trimer	Laccase from *Lentinus strigosus* 1566 (pH 7.2)	[11]
446	[M+H]^+^447(31%), 307(11%), 303(17%), 289(100%), 274(12%), 141(20%)	No info	Trimer		
340	[M+H]^+^341(100%), 323(42%), 311(17%), 208(76%)	No info	Dimer	Laccase from *Curvularia geniculata* VKM F-3561 (pH 7.2)	[31]
340	[M+H]^+^341(58%), 323(98%), 311(27%), 175(16%), 161(100%), 137(25%)	No info	Dimer		
588	no info	*β*-5/*β*-O-4	Trimer	Chemo-enzymatic pathway using laccase from *Trametes versicolor* (pH 4.5–5.0)	[61]
358	[M−H]^−^357(), 342(15%), 327(24%), 311(18%), 151(100%), 136(35%)	*β*-*β*	Dimer (pinoresinol)	Natural oligomers from wild-type poplar	[62]
358	[M−H]^−^357(), 342(25%), 339(100%), 327(25%), 221(22%), 203(15%), 191(5%)	*β*-5	Dimer		
376	[M−H]^−^375(), 357(6%), 327(98%), 195(15%), 179(3%), 165(4%)	*β*-O-4	Dimer		
572	[M−H]^−^571(), 553(12%), 523(100%), 391(29%), 375(4%), 343(20%), 327(3%), 195(), 179(11%)	*β*-O-4/*β*-O-4	Trimer		
584	[M−H]^−^583(), 565(17%), 535(100%), 387(2%), 369(53%), 357(23%), 195(3%)	*β*-5/*β*-O-4	Trimer		
584	[M−H]^−^583(), 565(23%), 535(100%), 387(42%), 373(11%), 357(14%), 343(5%), 195(4%)	*β*-*β*/*β*-O-4	Trimer		
618	[M−H]^−^617(), 587(5%), 569(100%), 491(18%), 403(35%), 391(5%), 195(1%)	*β*-5/*β*-O-4	Trimer		
**ferulic acid** (MW = 194 Da)					
**545**	[M+H]^+^546(54%), 528(100%), 510(17%), 366(18%)	No info	Dehydroderivative of trimer with [M+H]^+^548	Recombinant laccase or wild type laccase from *P. roridum* VKM F-3565 (pH 7.2)	present work, [41]
**547**	[M+H]^+^548(65%), 530(100%), 368(18%), 277(9%), 259(11%)	No info	Trimer		
636	[M+H]^+^637(50%), 581(100%)	No info	Trimer	Laccase from *Curvularia geniculata* VKM F-3561 (pH 7.2)	[31]
735	[M+H]^+^736(100%), 574(42%), 556(90%), 538(23%)	No info	Tetramer		
386	[M−H]^−^385(100%) ([M+H]^+^387)	*β*-5	Dimer	Laccase from *Trametes pubescens* strain CBS 696.94 (pH 5.0)	[63]
386	[M−H]^−^385(96%), 341(38%), 297(100%)	*β*-*β*	Dimer		
579	no info	No info	Trimer		
769	no info	No info	Tetramer		
324	[M+H]^+^325(87%), 307(100%), 223(94%)	No info	No info	Laccase (culture liquid) of *Myrothecium verrucaria* VKM F-3851 (pH 7.2)	[49]
386 (P1)	[M−H]^+^387(70%), 341(49%), 297(21%)	*β*-*β*	Dehydrodimer	Recombinant laccase from *Myceliophthora thermophila* (pH 7.5)	[64]
386 (P2)	[M−H]^+^387(75%), 343(45%), 297(59%)	*β*-5	Dehydrodimer		
386 (P3)	[M−H]^+^387(90%), 343(65%), 325(53%), 297(11%)	*β*-O-4	Dehydrodimer		
340 (P4)	[M−H]^+^341(92%), 323(23%), 235(8%), 177(80%)	*β*-5	Dehydrodimer		
403 (P5)	[M−H]^+^404(3%), 387(93%), 341(38%), 271(62%), 219(48%), 175(36%)	*β*-*β*	Dehydrodimer		
770 (P6)	[M−H]^+^771(92%), 753(100%), 717(27%)	No info	DehydroTetramer		
770 (P7)	[M−H]^+^771(100%), 735(45%), 689(20%)	No info	Dehydrotetramer		
298	[M−H]^−^297.111(-), 146(-), 109(-)	No info	Double decarboxylated diferulic acid	Laccases from *Pleurotus citrinopileatus* LGAM 28,684 (pH 4.0)	[65]
342	[M−H]^−^341(-), 281(-), 267(-), 209(-), 159(-), 146(-)	No info	Decarboxylated Diferulic acid		
386	[M−H]^−^385(-), 267(-), 239(-)	No info	Diferulic acid		
578	[M−H]^−^577(-), 193(-)	No info	Triferulic acid		
534	[M−H]^−^533(-), 193(-)	No info	Decarboxylated Triferulic acid		
566	[M−H]^−^565(-), 193(-), 178(-), 149(-), 134(-)	No info	Triferulic acid		
552	[M−H]^−^551(-), 193(-), 178(-), 149(-), 134(-)	No info	Decarboxylated Triferulic acid (water molecule missing)		
386 (P1)	[M−H]^−^385(43%), 341(100%), 326(24%), 282(8%), 193(16%)	*β*-4	Dimer	Laccase from *Pyricularia* *oryzae* (pH 6.0)	[66]
386 (P2)	[M−H]^−^385(16%), 341(68%), 297(7%), 281(15%), 173(42%), 159(73%), 123(100%)	*β*-O-4	Dimer		
358	no info	*β*-5	Dimer	Natural compounds from maize bran fiber	[67,68]
344	no info	5-5	Dimer		
578	no info	*β*-5/*β*-O-4	Trimer		
596	no info	5-5/*β*-O-4	Trimer		
578	[M−H]^−^577	5-5/*β*-O-4	Dehydrotrimer		
578	[M−H]^−^577	*β*-O-4/*β*-O-4	Dehydrotrimer	Natural compound from saponified maize bran	[69]
578	[M−H]^−^577	*β*-O-4/*β*-*β*(cyclic)	Dehydrotrimer		

## Data Availability

The original contributions presented in this study are included in the article/Supplementary Materials. Further inquiries can be directed to the corresponding author.

## Supplementary Materials

The following supporting information can be downloaded at: https://www.mdpi.com/article/10.3390/biom15101437/s1; Table S1: The scheme of purification of the recombinant laccase of *P. roridum* VKM F-3565 from the culture liquid of *K. phaffii* GS115; Figure S1: The expression vector pET22b-F3565 for heterologous expression of the laccase from *P. roridum* VKM F-3565 in *E. coli* BL21 (DE3) cells; Figure S2: The linearized expression vector pPIC9K-F-3565 for heterologous expression of the laccase from *P. roridum* VKM F-3565 in *K. phaffii* GS115 cells; Figure S3: Connolly accessible surface representations colored according to electrostatic potential (−5 keV, red; +5 keV, blue) for the laccase of *P. roridum* VKM F3565 at different pH. The blue circle marks the entrance to the T1 center of the laccase, and the green circle marks the entrance to the proton channel.
