# Supplementary material for: Specific Phenylpropanoid Oligomerization in a Neutral Environment by the Recombinant Alkaline Laccase from Paramyrothecium roridum VKM F-3565
Source: Biomolecules. 2025 Oct 11;15(10):1437. doi: 10.3390/biom15101437 (PMC12563105; doi:10.3390/biom15101437)
Supplement: Supplementary file 1 [file biomolecules-15-01437-s001.zip › biomolecules-3926021-supplementary.pdf]

Supplementary materials

Table S1. The scheme of purification of the recombinant laccase of *P. roridum* VKM F-3565 from the culture liquid of *K. phaffii* GS115.

Purification step	Total protein, mg	Specific activity, U/mg	Total activity, U	Purification, fold*	Yield, %**
Culture liquid	319.8	4.54	1451.89	1.00	100.00
DEAE-Toyopearl	108.4	15.05	1631.42	3.32	112.36
Q-Sepharose	41.25	36.45	1503.56	8.03	103.56
Resource Q	20.03	71.50	1432.15	15.75	98.64

* - The purification fold was calculated by the ratio of the specific activity after each purification step versus the specific activity of the culture liquid

** - The yield of laccase at each stage of purification was calculated as a percentage of the total laccase activity in the culture liquid, taken as 100%

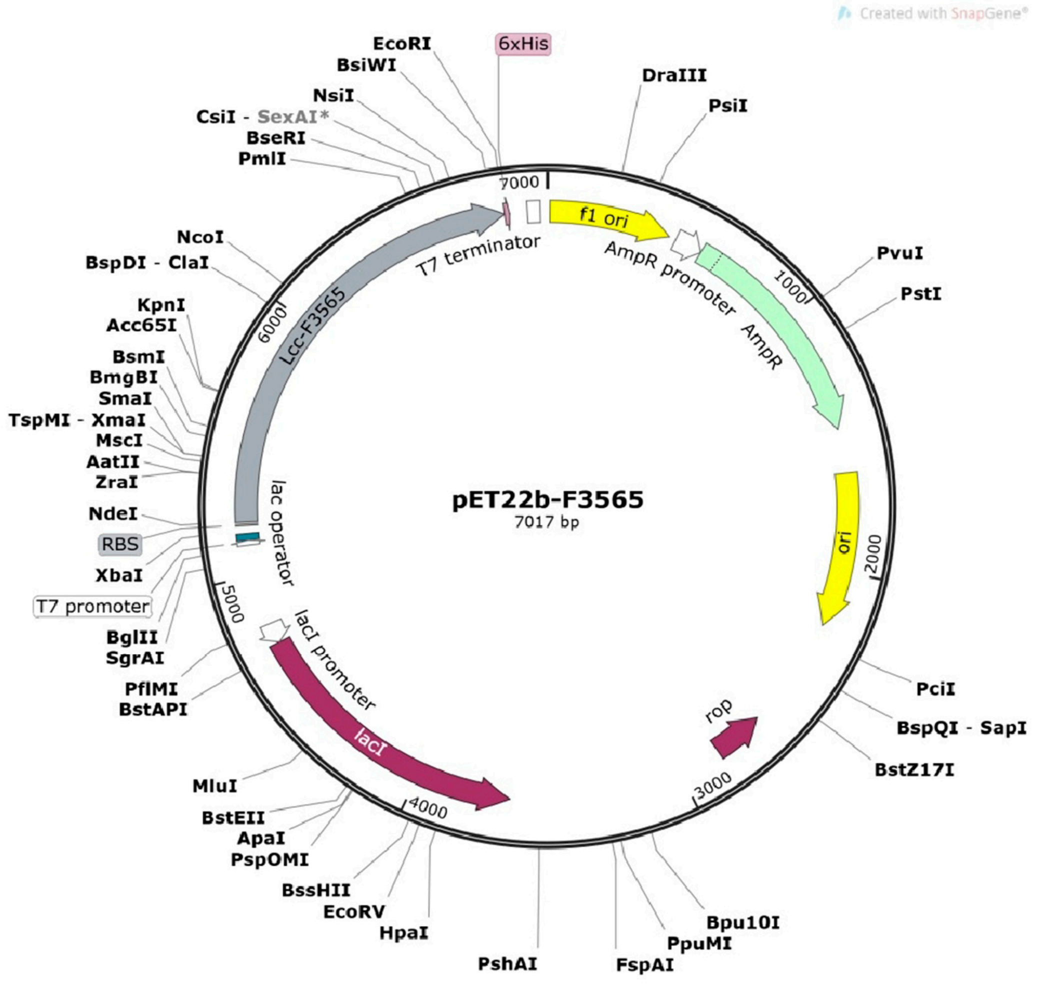

Figure S1. The expression vector pET22b-F3565 for heterologous expression of the laccase from *P. roridum* VKM F-3565 in *E. coli* BL21 (DE3) cells

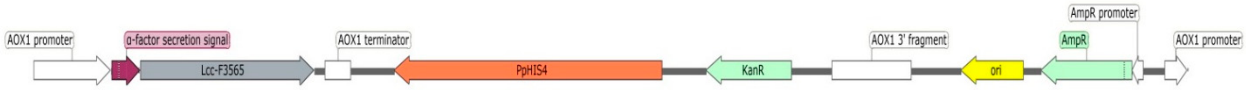

Figure S2. The linearized expression vector pPIC9K-F-3565 for heterologous expression of the laccase from *P. roridum* VKM F-3565 in *K. phaffii* GS115 cells

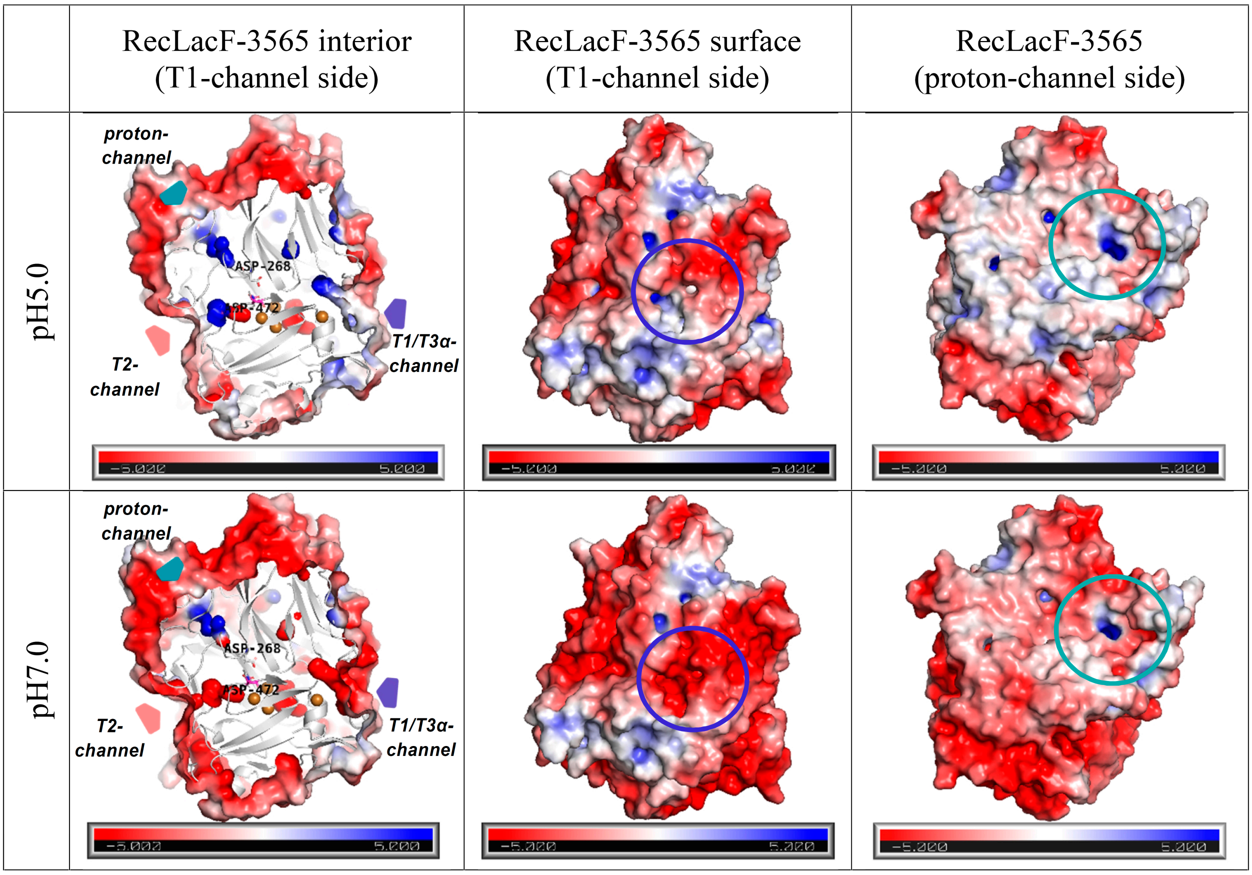

Figure S3. Connolly accessible surface representations colored according to electrostatic potential (-5 keV, red; +5 keV, blue) for the laccase of *P. roridum* VKM F3565 at different pH. The blue circle marks the entrance to the T1 center of the laccase, and the green circle marks the entrance to the proton channel.
